# Recent advances in the management of penile cancer

**DOI:** 10.12688/f1000research.18185.1

**Published:** 2019-04-26

**Authors:** Maximilian J Johnston, Raj Nigam

**Affiliations:** 1 Department of Urology, Royal Surrey County Hospital, Guildford, UK; 2 Department of Surgery & Cancer, Imperial College London, London, UK; 3 Institute of Urology, University College London Hospitals, London, UK

**Keywords:** penile, PeIN, cancer, metastasis, HPV

## Abstract

Penile cancer is a rare condition and can be very complex to manage. Advances in surgical techniques, imaging, pathological classification and patient pathways have led to improved patient care. The diagnosis of pre-malignant change, penile cancer and metastatic disease along with advances in their treatment are detailed in this review which aims to update clinicians from multiple specialties and countries on penile cancer.

## Background

Penile cancer (PC) is a rare condition affecting fewer than 1:100,000 European males ^1^. Traditionally, PC was managed with aggressive, radical surgery to achieve satisfactory oncological results. These treatments resulted in significant clinical and psychological morbidity ^2^. This, however, is changing. PC is one of the few cancers in which the TNM (tumour, node, metastasis) staging criteria have changed multiple times in recent years (see Table 1 for the eighth edition) ^3^. The reason for this is the rapid advancement in diagnostic and therapeutic strategies for PC. In this narrative review, we outline recent advances in the treatment of this condition to update clinicians from multiple specialties and countries. To inform this review, a Medline search for articles containing the terms “penile cancer”, “penile intraepithelial neoplasia”, and “carcinoma of the penis” was carried out, and the identified literature is summarised below.

**Table 1.** The eighth edition of the tumour-node-metastasis staging classification for penile cancer.

Primary tumour (pT)	
Tx	Primary tumour cannot be assessed
T0	No evidence of primary tumour
Tis	Carcinoma *in situ* (penile intraepithelial neoplasia)
Ta	Non-invasive localised squamous cell carcinoma
T1	Tumour invades subepithelial connective tissue, dermis or lamina propria
T1a	Tumour is without lymphovascular invasion or perineural invasion and is not high-grade
T1b	Tumour exhibits lymphovascular invasion or perineural invasion (or both) or is high-grade
T2	Tumour invades corpus spongiosum with or without urethral invasion
T3	Tumour invades corpora cavernosum with or without urethral invasion
T4	Tumour invades other adjacent structures
Regional lymph nodes (pN)	
Nx	Lymph node metastasis cannot be established
N0	No lymph node metastasis
N1	Not more than two unilateral inguinal metastases, no extranodal extension
N2	At least three unilateral inguinal metastases or bilateral metastases
N3	Extranodal extension of lymph node metastasis or pelvic lymph node metastases
Distant metastasis (M)	
M0	No distant metastasis
M1	Distant metastasis present

## Diagnosis of pre-malignant changes and penile cancer

**Figure 1.**
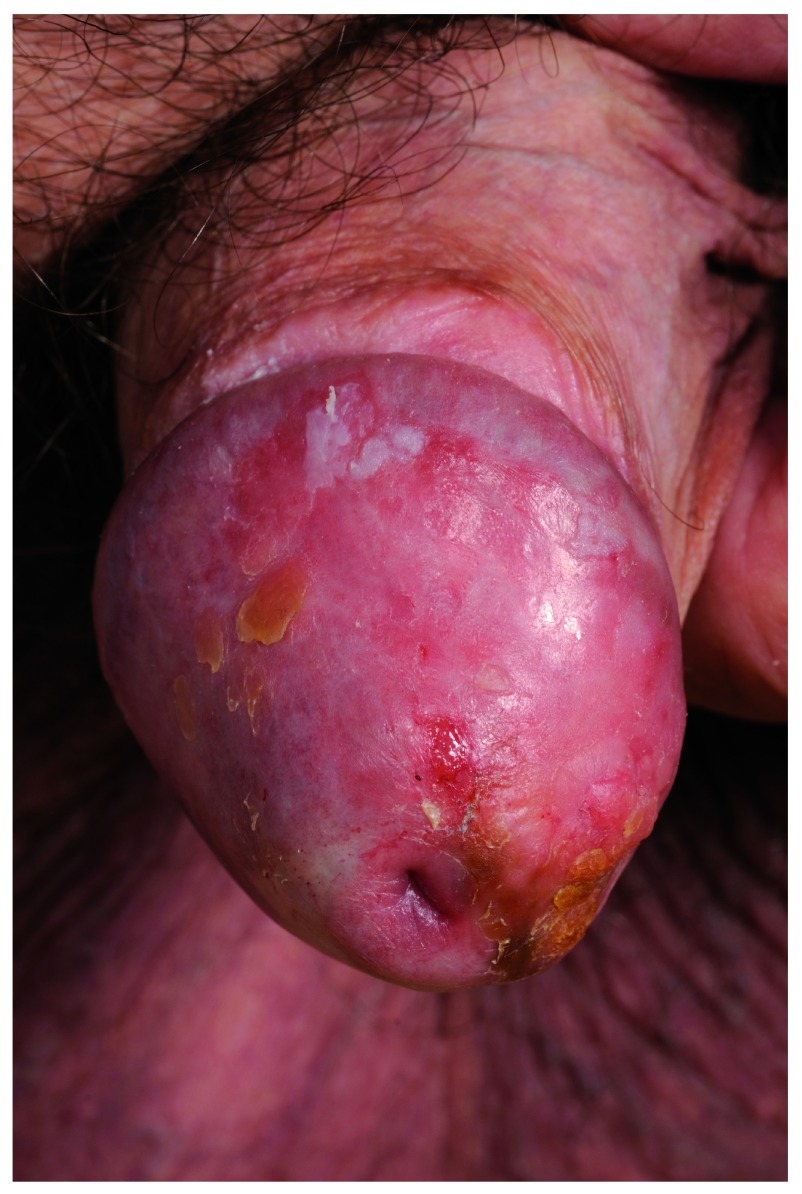
Penile intraepithelial neoplasia of the glans penis. Used with permission from The Royal Surrey County Hospital.

**Figure 2.**
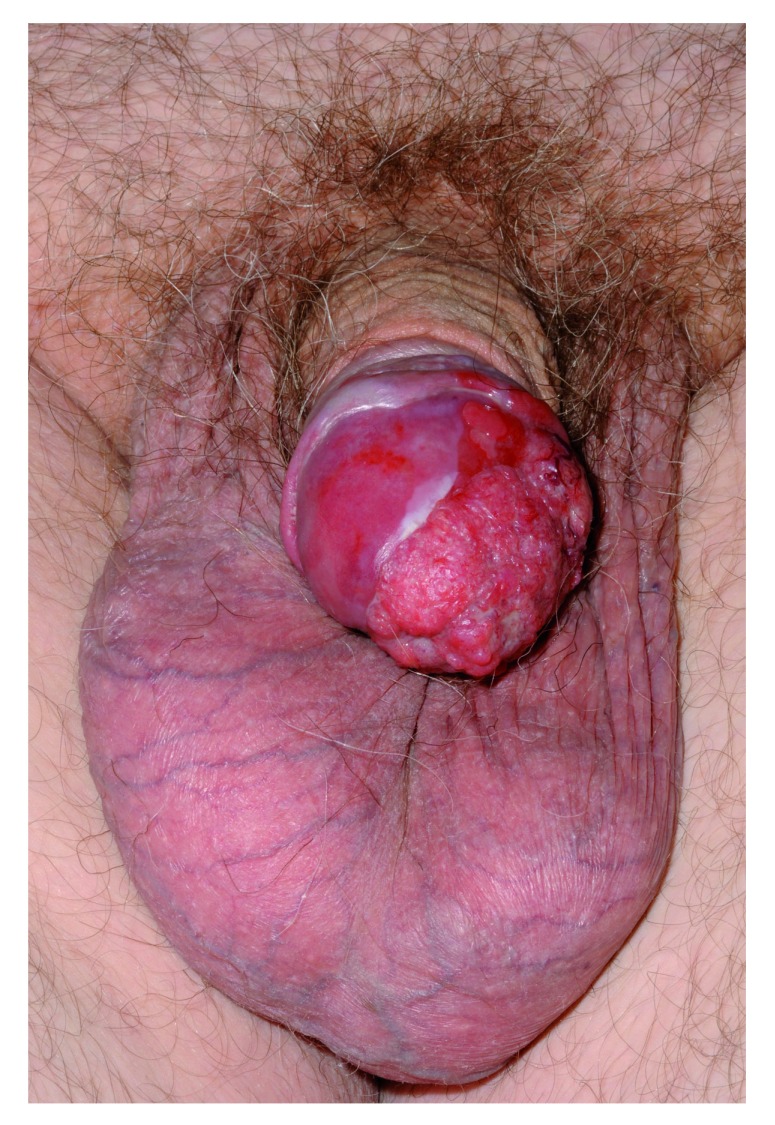
Squamous cell carcinoma of the penis involving the prepuce, glans and urethral meatus. Used with permission from The Royal Surrey County Hospital.

**Figure 3.**
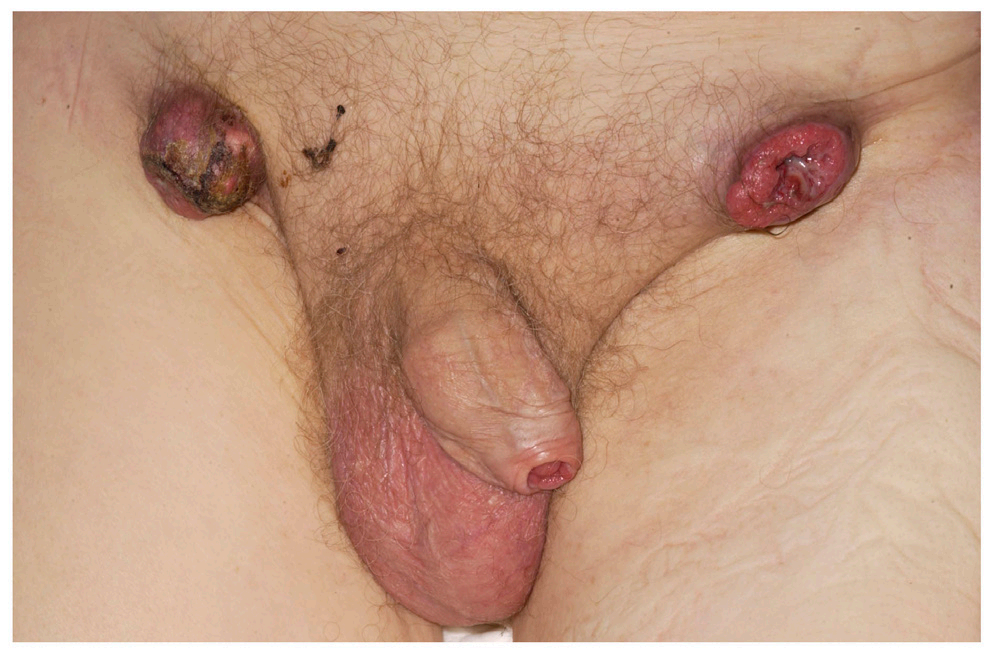
Bilateral fungating inguinal metastases with primary visible under phimotic skin. Used with permission from The Royal Surrey County Hospital.

The diagnosis of PC and pre-malignant change (penile intraepithelial neoplasia, or PeIN) is increasing, (see Figure 1 and Figure 2). First, this is due to a higher number of biopsies being performed aiming to pick up these conditions. This phenomenon can be ascribed partly to increased public awareness. Charities and websites aiming to promote men’s health (for example, the Movember Foundation and the Orchid charity) along with a generational change in attitudes have led to more men presenting to a doctor for conditions affecting the genitalia. A culture shift has made it more acceptable for men to self-examine and present early to a healthcare professional. Despite this, recent research has shown that awareness of and access to information regarding PC are still poor ^4^. This can result in late presentation and poor prognosis (see Figure 3).

Second, there are more biopsies of the penis being carried out by urologists, dermatologists and sexual health physicians as awareness in the primary and secondary care setting increases in response to published guidance from June 2015 by the National Institute for Health and Care Excellence in the UK ( https://www.nice.org.uk/guidance/ng12). These biopsies, often performed at the same time as a circumcision for troublesome phimosis, can be performed under local anaesthetic.

Lastly, the association between human papilloma virus (HPV) and PC has come under the spotlight in recent years. The link between HPV and cervical cancer is well established ^5^, and many countries have initiated vaccination programmes for females prior to exposure to sexual activity. Men, however, are not routinely vaccinated in most countries, although recent guidance in the UK has recommended it ^6^. Whilst the prevalence of HPV infection in patients with PC, or indeed those with PeIN changes, varies from 20 to 60%, the rate is much greater in females with cervical cancer (93%) ^5, 7, 8^.

This discrepancy is explained by the proposal that there are two principal pathways that lead to the development of PC: the HPV pathway, caused by HPV infection (the implicated strains are 16, 18, 31, 33, 45, 56, and 65 ^7^), and the lichen sclerosus pathway. The latter will not be affected by an HPV vaccination programme. This has caused controversy recently, and arguments both for and against male vaccination have been made within the literature and the media; one of the principal arguments against is cost-effectiveness ^9^.

The pathological classification distinguishing HPV-related undifferentiated and non-HPV differentiated PeIN should allow more targeted vaccination of males in the future ^10, 11^. Promising research into immunotherapeutic treatments aiming to target specific areas of the HPV pathway with T-cell therapy in combination with differing chemotherapeutic regimens is ongoing ^12^.

In recent years, the management of PeIN has changed insofar as the recommendation that patients with this condition undergo surgical treatment (circumcision, wide local excision and glans resurfacing) if a course of topical chemotherapy has failed. In some cases, initial treatment is surgical to reduce recurrence rates ^13^. The reason for this is to prevent further cellular damage by HPV to preputial skin by removing it and this was proposed in response to poor efficacy of topical agents ^14, 15^.

## Diagnosis of metastatic disease

Emerging strategies for the diagnosis of metastatic disease have recently been developed and implemented. The most impactful change in this area has been the use of dynamic sentinel lymph node sampling for the diagnosis of impalpable disease within inguinal lymph nodes. This technique is prefaced on the recognised stepwise progression of PC, which starts at the inguinal nodes as the first site of metastasis. Although this technique was conceived in the 1970s, it is only with the introduction of the use of pigment and lymphoscintigraphy that the benefits of this technique over more traditional radical inguinal lymph node dissection have become clear ^16^. The development of advanced radiology techniques continues apace, and the use of single-photon emission computed tomography (SPECT) is exciting. Initial studies show that the use of SPECT in addition to lymphoscintigraphy may improve the rate of detection of PC-infiltrated lymph nodes and accurately show their location ^17^. Further along the metastatic path, the use of positron emission tomography–computed tomography (PET-CT) has been shown to add value when managing patients with clinically suspicious groins or pelvis lymphadenopathy. In patients with clinically suspicious groins, the sensitivity and specificity of fluorodeoxyglucose (FDG) PET-CT were shown to be 96% and 100%, respectively ^18^. This is vital because accurate staging of patients is required to allow decisions to be made regarding oncological treatment. The use of magnetic resonance imaging PET is currently being trialled; potential advantages include the procurement of both geographical and functional information regarding PC.

The ability to operatively target the sentinel node allows PC surgeons to reliably diagnose and treat metastatic disease whilst avoiding the significant morbidity of groin dissection in certain patients ^19^. Patients undergoing dynamic sentinel node sampling are able to undergo a day surgery procedure through excellent teamwork between nuclear medicine radiologists and surgeons whereas those undergoing groin dissection typically have a much longer length of stay ^20^. Other advances in this area include the use of minimally invasive videoendoscopic techniques ^21^. Some of the proposed advantages of these techniques include smaller wounds, preservation of saphenous and femoral vasculature, and satisfactory lymph node yields; it is likely that these techniques will increase in coming years ^22, 23^.

There are other changes that have taken place in the diagnosis of metastatic PC and these include the alteration in philosophy of clinicians regarding palpable nodes. Previously, swollen and palpable inguinal nodes were observed for 6 weeks to allow the possibility of lymphadenopathy secondary to infection (many cases of PC have superadded infection) to settle. We now know that this could lead to a critical delay in the treatment of PC. Palpable nodes are now rapidly investigated with CT scanning and ultrasound-guided fine-needle aspiration cytology or biopsy ^19^.

Lastly, the combination of highly accurate magnetic resonance imaging with intracavernosal injection of prostaglandin E _1_ to produce an artificial erection has allowed much more accurate staging of primary tumours. Although the assessment of urethral invasion in PC remains challenging, the sensitivity and specificity of this technique for invasion of the tunica albuginea are satisfactory at 82.1% and 73.6%, respectively ^24^. This is also a particularly useful technique in assessing the presence of skip lesions in the corpora cavernosa, thus allowing accurate and correct management of the primary lesion.

These advances in diagnostic techniques have allowed accurate staging of tumours and improved joint decision-making for patients with PC. However, one of the major changes in the management of PC is the recent development of supraregional networks for PC, pioneered in the UK. In the mid-2000s, the National Institute for Clinical Excellence (now part of the National Institute for Health and Care Excellence) recommended that PC management be centralised into centres with significant expertise in the management of this rare condition. At the time the supraregional networks were conceived, the typical urologist was seeing one or two PC patients per year, which caused considerable variation in practice ^25^. This was unpalatable because both under- and over-treatment of PC can have life-changing or life-ending consequences. The development of these networks is thought to have improved the speed of PC diagnosis and treatment pathway along with concentrating knowledge and experience in a limited number of large institutions aiming for clinical excellence ^26^. In the time these networks have been around, the cancer-specific survival of 203 patients with PC was shown to be 85% ^27^. Similar programmes are under way in other European countries following the incorporation of recommendations regarding PC treatment in high-volume centres from the European Association of Urology ^15^.

## Management of the primary lesion

The most crucial advance in surgery for PC has been the principle of tissue preservation and reconstructive techniques without oncological compromise. This has revolutionised the lives of PC patients who can avoid the clinical and psychological morbidity of more radical surgery and radiotherapy treatment modalities. A resection margin of more than 2 cm used to be required; however, less than 5 mm is now considered adequate to be oncologically safe for patients ^1^, dramatically increasing the number of organ-sparing procedures carried out ^28^.

Procedures such as glansectomy with split-thickness skin grafting and partial penile amputation with urethral centralisation have allowed good oncological disease control with improved functional and cosmetic outcomes for patients ^29^. Techniques such as urethral centralisation and neo-glans formation following partial penectomy are examples of further attempts to reduce the psychological morbidity of partial penectomy by recreating a glans and placing the urethra in a location to mimic the original anatomy prior to surgery ^30^. An example of a more radical technique, that still aims to facilitate later reconstruction once oncological control is certain, is total phallic reconstruction. For younger men with PC undergoing radical surgery, the urethra can be exteriorised to the pubic region, facilitating later reconstruction ^31^.

With these improvements in surgical technique and centralisation of PC care has come a greater pathological understanding of the disease. This has allowed PC surgeons to elucidate the risk factors for local recurrence following conservative surgery. These include perineural invasion, carcinoma *in situ*, positive margins and high-grade disease. This in turn allows tailored follow-up for patients with PC, improving the overall quality of their care ^32^.

## Advances in the management of metastatic disease

Building on the technique of dynamic sentinel node biopsy has allowed fewer radical groin dissections to be required for patients with PC, thereby reducing morbidity without oncological compromise. However, for many, radical inguinal lymph node dissection is still required. Careful understanding of the fascial planes and their preservation facilitates good wound closure and prevents the worst of wound complications, formerly the scourge of radical dissection. In addition, some dissections are performed unilaterally for proven metastatic disease, which in turn reduces morbidity. Currently, the use of radiotherapy for nodal metastases is not recommended ^33^. However, emerging research suggests that radiotherapy can improve regional control in PC patients with involved pelvic lymph nodes ^34^ without extranodal extension ^35^.

Pelvic lymph node dissection is still controversial, as it may not improve survival in low-risk disease but can in high-risk disease ^36^. However, as this can now be performed using minimally invasive techniques, the previous extended length of stay, wound morbidity and lymphoedema rates have all decreased. The use of robotic assistance for pelvic lymph node dissection may reduce complication rates further ^36^.

**Figure 4.**
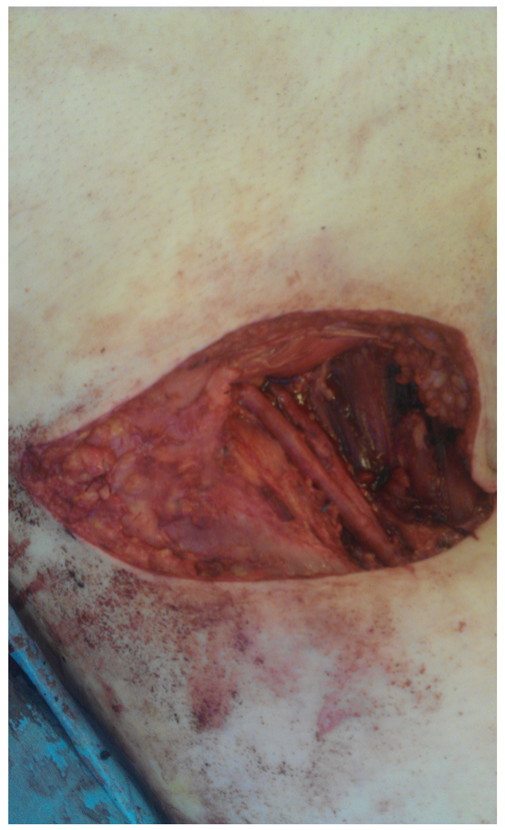
Exposure following radical inguinal lymph node dissection. Used with permission from The Royal Surrey County Hospital.

For more advanced disease, the use of vertical rectus abdominis myocutaneous, tensor fascia lata and gracilis flaps (which carry their own blood supply) is now feasible and allows coverage of large defects following extensive surgery (see Figure 4– Figure 6). Although this is debilitating surgery, it can have a positive impact on a palliative patient’s quality of life and is an important advance ^37– 39^.

**Figure 5.**
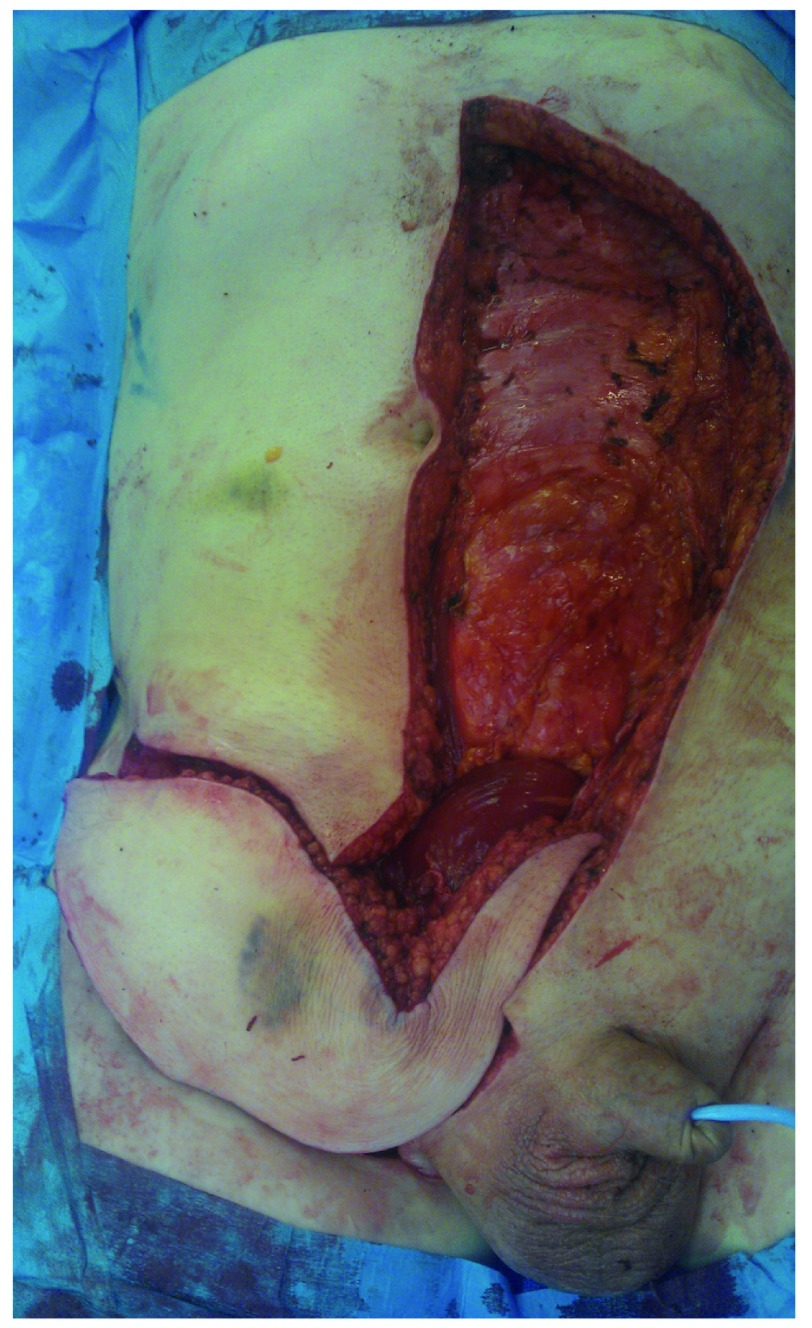
VRAM flap rotated on inferior epigastric pedicle to cover defect. Used with permission from The Royal Surrey County Hospital.

**Figure 6.**
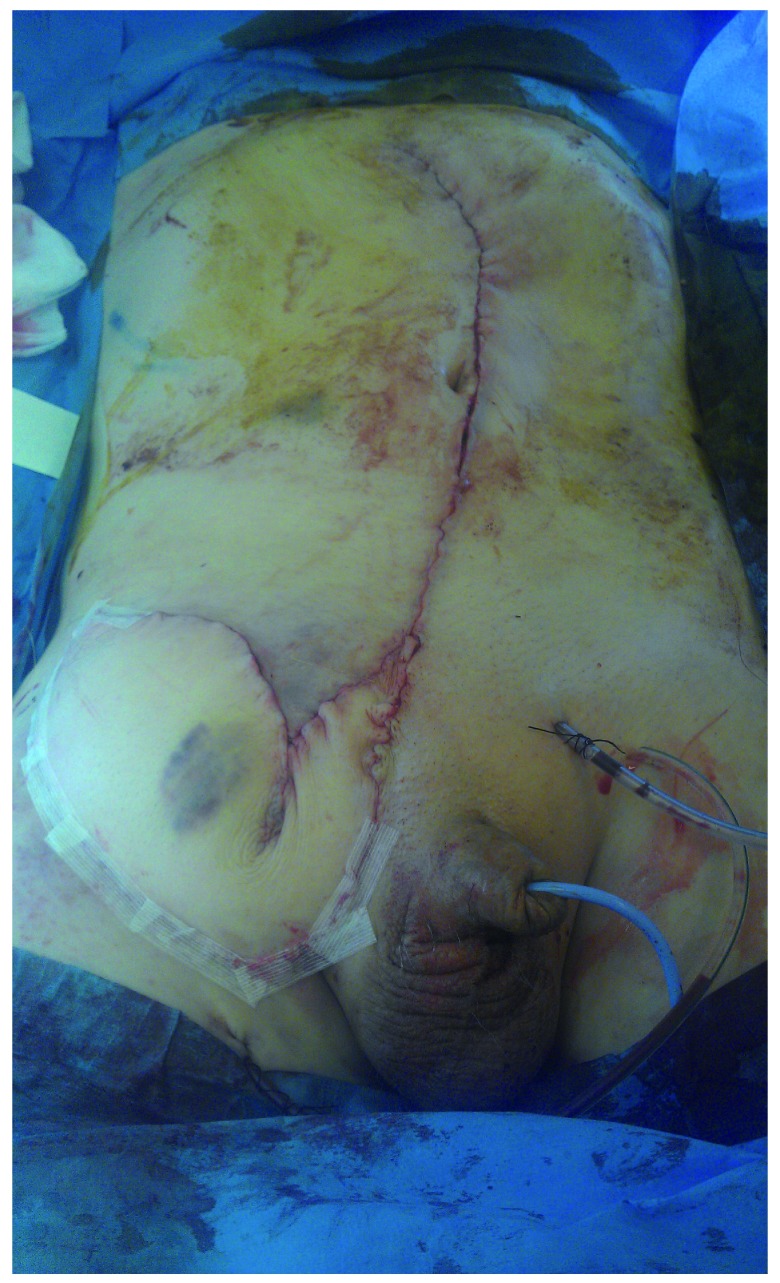
Closure with percutaneous drain *in situ*. Used with permission from The Royal Surrey County Hospital.

## Conclusions

This article describes the recent advances in the diagnosis and management of PC. It explains where practice-changing research has occurred and discusses areas of controversy. Increased knowledge and research are allowing men with PC to have improved functional and oncological outcomes.
